# Evidence for biochemical barrier restoration: Topical solenopsin analogs improve inflammation and acanthosis in the KC-Tie2 mouse model of psoriasis

**DOI:** 10.1038/s41598-017-10580-y

**Published:** 2017-09-11

**Authors:** Jack L. Arbiser, Ron Nowak, Kellie Michaels, Yuliya Skabytska, Tilo Biedermann, Monica J. Lewis, Michael Y. Bonner, Shikha Rao, Linda C. Gilbert, Nabiha Yusuf, Isabella Karlsson, Yi Fritz, Nicole L. Ward

**Affiliations:** 1 Department of Dermatology, Emory University School of Medicine, Atlanta, GA 30322 USA; 2 Veterans Affairs Medical Center, Decatur, GA 30322 USA; 3 Case Western Reserve University, Cleveland, OH 44106 USA; 4 Department of Dermatology and Allergology Technische Universität München, München, Germany; 5 Department of Dermatology, University of Alabama, Birmingham, AL 35294 USA

## Abstract

Psoriasis is a chronic inflammatory skin disease affecting 2.5–6 million patients in the United States. The cause of psoriasis remains unknown. Previous human and animal studies suggest that patients with a susceptible genetic background and some stimulus, such as barrier disruption, leads to a coordinated signaling events involving cytokines between keratinocytes, endothelial cells, T cells, macrophages and dendritic cells. Ceramides are endogenous skin lipids essential for maintaining skin barrier function and loss of ceramides may underlie inflammatory and premalignant skin. Ceramides act as a double-edged sword, promoting normal skin homeostasis in the native state, but can be metabolized to sphingosine-1-phosphate (S1P), linked to inflammation and tumorigenesis. To overcome this difficulty, we synthesized solenopsin analogs which biochemically act as ceramides, but cannot be metabolized to S1P. We assess their *in vivo* bioactivity in a well-established mouse model of psoriasis, the KC-Tie2 mouse. Topical solenopsin derivatives normalized cutaneous hyperplasia in this model, decreased T cell infiltration, interleukin (IL)-22 transcription, and reversed the upregulation of calprotectin and Toll-like receptor (TLR) 4 in inflamed skin. Finally, they stimulated interleukin (IL)-12 production in skin dendritic cells. Thus suggesting barrier restoration has both a biochemical and physical component, and both are necessary for optimal barrier restoration.

## Introduction

Psoriasis is a common autoimmune disease mediated in part by interleukin (IL) 17-biased T lymphocytes^1, 2^. It affects 1–2% of the US population and the mainstays of treatment are topical agents for limited disease and systemic and biologic agents for more generalized disease. Systemic agents include methotrexate, vitamin A derivatives and cyclosporine, while biologic therapies target tumor necrosis factor alpha (TNFα), IL-23 and IL-17. Biologic therapies have made a major impact on the quality of life of patients with severe psoriasis, but are expensive and predispose patients to infection and malignancy^3–5^. Finally, biologic agents are not indicated for the majority of patients who have mild to moderate disease. For decades, the mainstay of topical treatment for psoriasis was topical steroids. While effective in a significant number of patients, topical steroids are not a panacea for psoriasis. First, resistance (tachyphylaxis) is observed after long-term use. Second, cutaneous atrophy is a well-known side effect of long-term topical steroid use^6^. Finally, some lesions are highly refractory to topical steroids, especially thicker lesions. While new topical agents have been introduced, none have significantly impacted the rate of use of topical steroids. These agents include vitamin D analogs, retinoids, and calcineurin inhibitors. Barrier restoration with emollients contributes to resolution of psoriasis, but does not fully reverse the psoriatic phenotype.

Ceramides play an essential role in maintaining the barrier function of the skin and loss of barrier function increases expression of vascular endothelial growth factor (VEGF), a major mediator of angiogenesis and inflammation^7^. However, the failure of emollient barriers to fully reduce inflammation implies a specific biochemical role of ceramides in epidermal homeostasis. Ceramides are generated from lipid precursors and can be degraded by enzymatic hydrolysis or metabolized into sphingosine-1-phosphate (S1P), a mediator of cellular growth, carcinogenesis, and inflammation^8^. We have synthesized ceramide analogs that are incapable of hydrolysis or metabolism to S1P and have demonstrated that these analogs have *in vivo* efficacy in a well-validated murine model of psoriasis^9^. Gene array analysis validated by immunohistochemistry indicates that these analogs decrease production of S100A8 and S100A9 (calprotectin) and the Toll-like receptor (TLR) 4 calprotectin receptor, leading to a reduction in epidermal thickness and lymphocytic infiltration^10^. Calprotectin is a dimer of S100A8 and S100A9, which complexes both calcium and zinc, and may act as a pro-inflammatory factor through the sequestration of zinc. Fecal calprotectin is now used as a biomarker of colonic inflammation^11–14^. Gene array of treated vs. untreated inflamed skin shows downregulation of IL-22 and upregulation of AP-1 members FosB and c-Jun. Calprotectin has been implicated as the human PSORS4 locus^15, 16^. Given that IL-22 plays a major role in mediating both inflammation and carcinogenesis^17–20^, these compounds could potentially be useful in a wide variety of skin disorders. Thus, restoration of barrier function with stable ceramide analogs leads to an upregulation of IL-12, downregulation of IL-22 and stable production of IL-23, leading to an antiangiogenic phenotype. Finally, we introduce the concept of biochemical barrier restoration that explains, in part, why physical barrier restoration is, by itself, incapable of fully resolving cutaneous inflammation.

## Results

### Topical S12 and S14 treatment of KC-Tie2 mice decreases epidermal thickness and immune cell infiltrate

**Figure 1:**
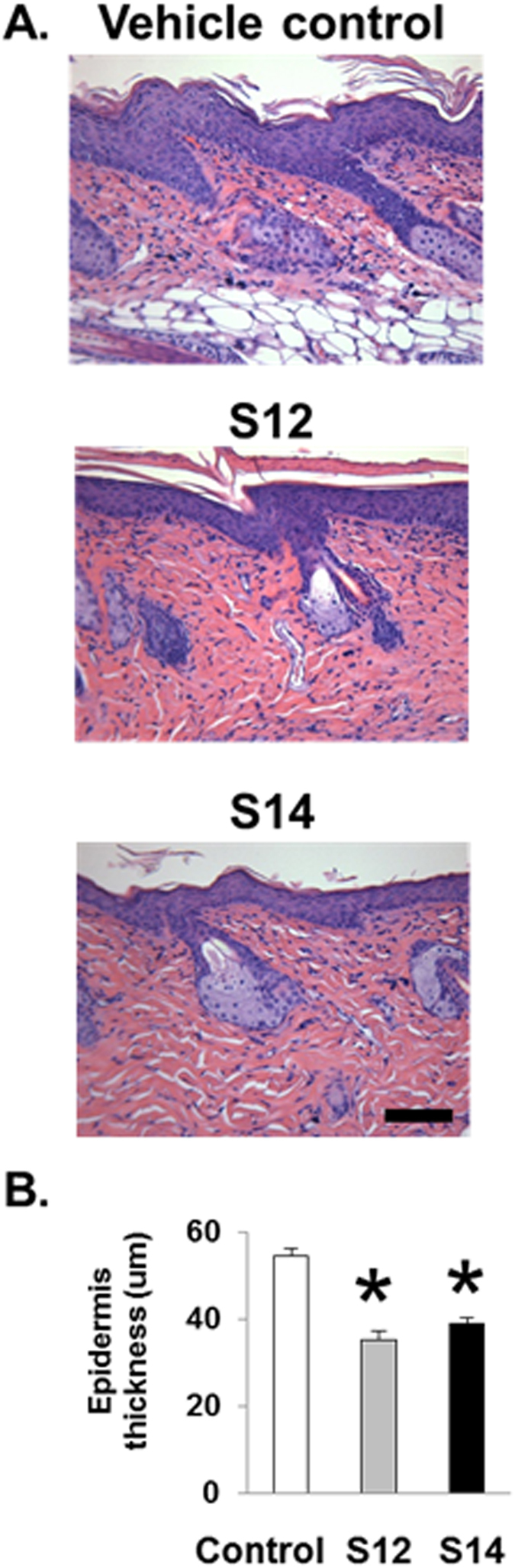
Treatment of inKC-Tie2 mice with S12 and S14 results in decreased epidermal thickness (acanthosis). (**A**) Representative images of H&E-stained dorsal skin sections from KC-Tie2 mice following treatment with S12, S14 or vehicle cream. (**B**) Quantification of epidermal thickness (μm) of H&E-stained dorsal skin sections of vehicle treated (n = 8), S12 (n = 9) and S14 (n = 9) KC-Tie2 mice. Values shown represent the mean ± SEM. Data were analyzed using a Student’s t-test. P values are as indicated. Scale bar = 50 μm.

S12 and S14 are solenopsin analogs that have 12 and 14 carbon side chains respectively, on the piperidine pharmacophore. KC-Tie2 mice treated with either S12 or S14 for 28 days showed significant decreases (n = 9/grp; 28–35%; P < 0.001) in acanthosis and hyperkeratosis compared to animals treated with vehicle control (n = 8; Fig. 1). This pattern of acanthosis and hyperkeratosis is highly similar to the pathologic changes seen in human psoriasis.

**Figure 2:**
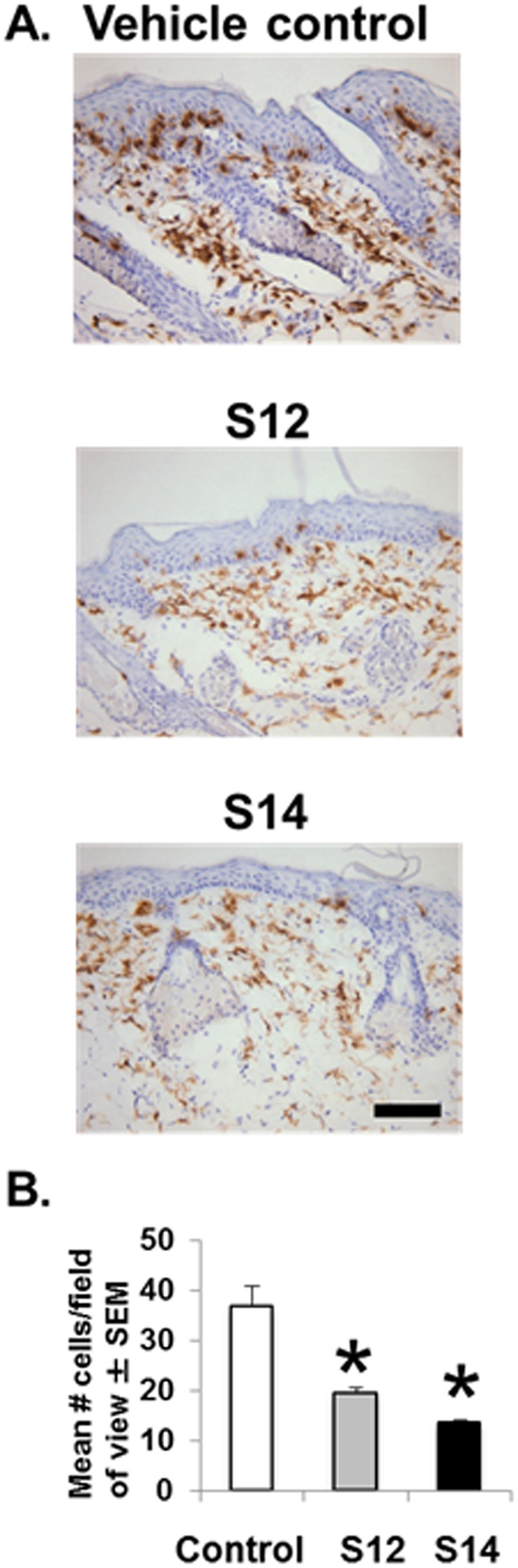
Treatment of CD4+ T cells decrease in KC-Tie2 mice with topical S12 and S14 analogs results in decreased CD4+ infiltration. (**A**) Representative images of CD4+ stained dorsal skin sections from KC-Tie2 mice following treatment with S12, S14 or vehicle cream. (**B**) Quantification of CD4+ T cell numbers in KC-Tie2 mice treated with vehicle (n = 8), S12 (n = 9) and S14 (n = 9). Values shown represent the mean # of cells/FOV (field of view) ± SEM. Data were analyzed using a Student’s t-test. P values are as indicated. Scale bar = 50 μm.

**Figure 3:**
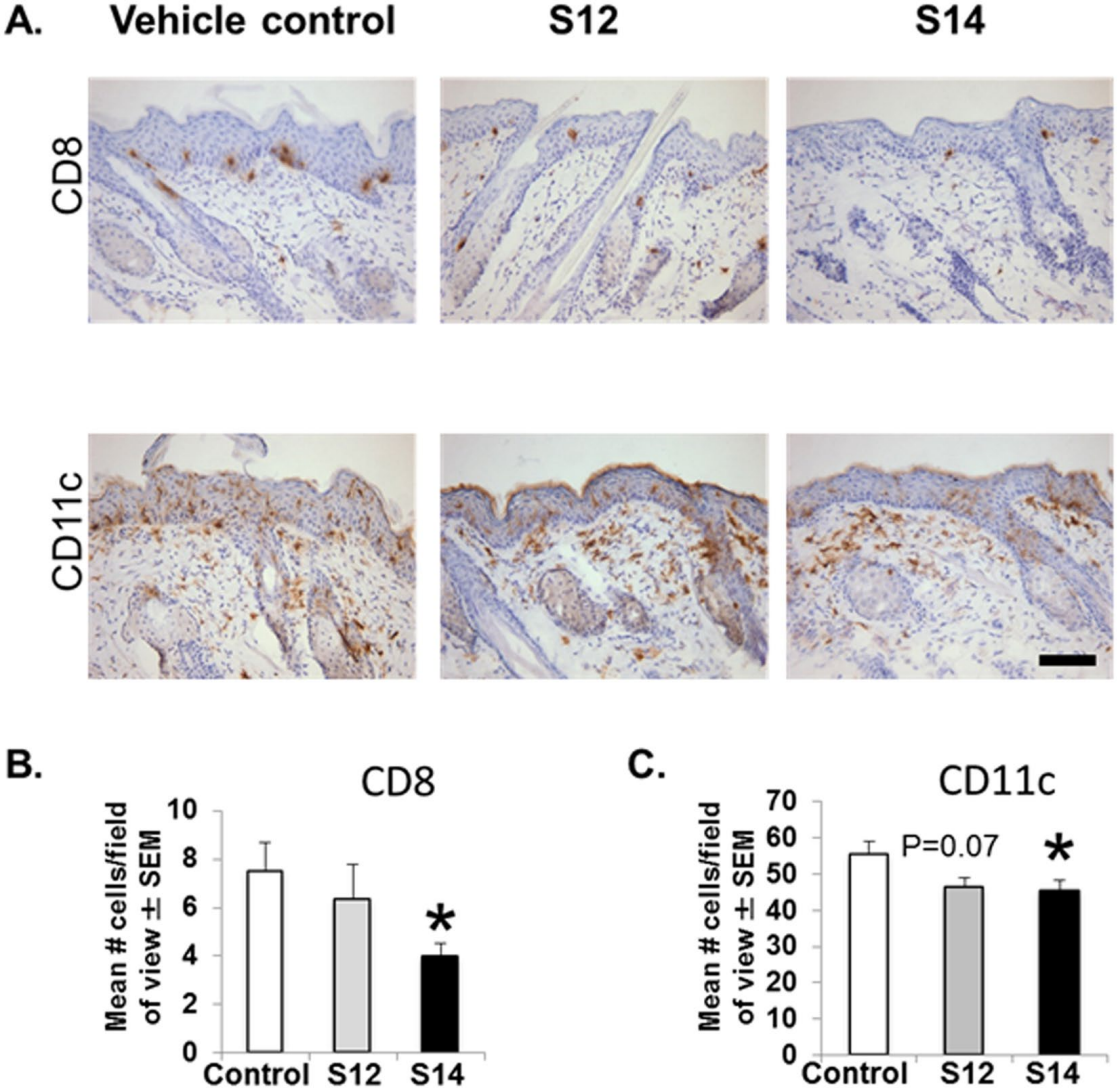
S14 causes decreased infiltration of CD8+ and CD11c+ cells compared with S12. (**A**) Representative images of CD8+ stained and CD11c+ stained dorsal skin sections from KC-Tie2 mice following treatment with S12, S14 or vehicle cream. (**B**) Quantification of CD8+ T cell and (**C**). Cd11c+ cell numbers in KC-Tie2 mice treated with vehicle (n = 8), S12 (n = 9) or S14 (n = 9). Values shown represent the mean # of cells/FOV (field of view) ± SEM. Data were analyzed using a Student’s t-test. P values are as indicated. Scale bar = 50 μm.

Psoriasis is characterized by T cell infiltration. Significant decrease in CD4+ T cell infiltration (47–63%; P < 0.001) accompanied the improvement in acanthosis in both S12- and S14-treated mice (Fig. 2). In order to determine whether solenopsin analogs had differential effect on T cell and murine dendritic cells (DC), we examined the effect of treatment on KC-Tie2 mice treated with S14, but not S12. Treatment with S14 resulted in significant decreases in CD8+ T cell (47%; P < 0.01) and CD11c+ dendritic cell infiltration into dorsal skin (18%; P = 0.04) compared to vehicle controls (Fig. 3). It may be that the 14 carbon chain is an optimal length to penetrate cell membranes and inhibit the function of both CD8+ and CD11c+ dendritic cells. We have made analogs with longer side chains than 14 carbons and have found them to be not as effective^9^.

In order to gain insight into the mechanism of the efficacy of solenopsin analogs, RNA was generated from vehicle- and compound-treated skin and subjected to gene array analysis. The heat maps showed strong overlap between the genes regulated by S12 and S14 compared with controls (Figure S1) and genes commonly regulated by both analogs compared with vehicles were studied further. These include specific AP-1 subunits, including FosB and ATF3.

### S12 and S14 compounds cause a decrease in IL-22, an increase in IL-12, but no changes in IL-23 production by dendritic cells

**Figure 4:**
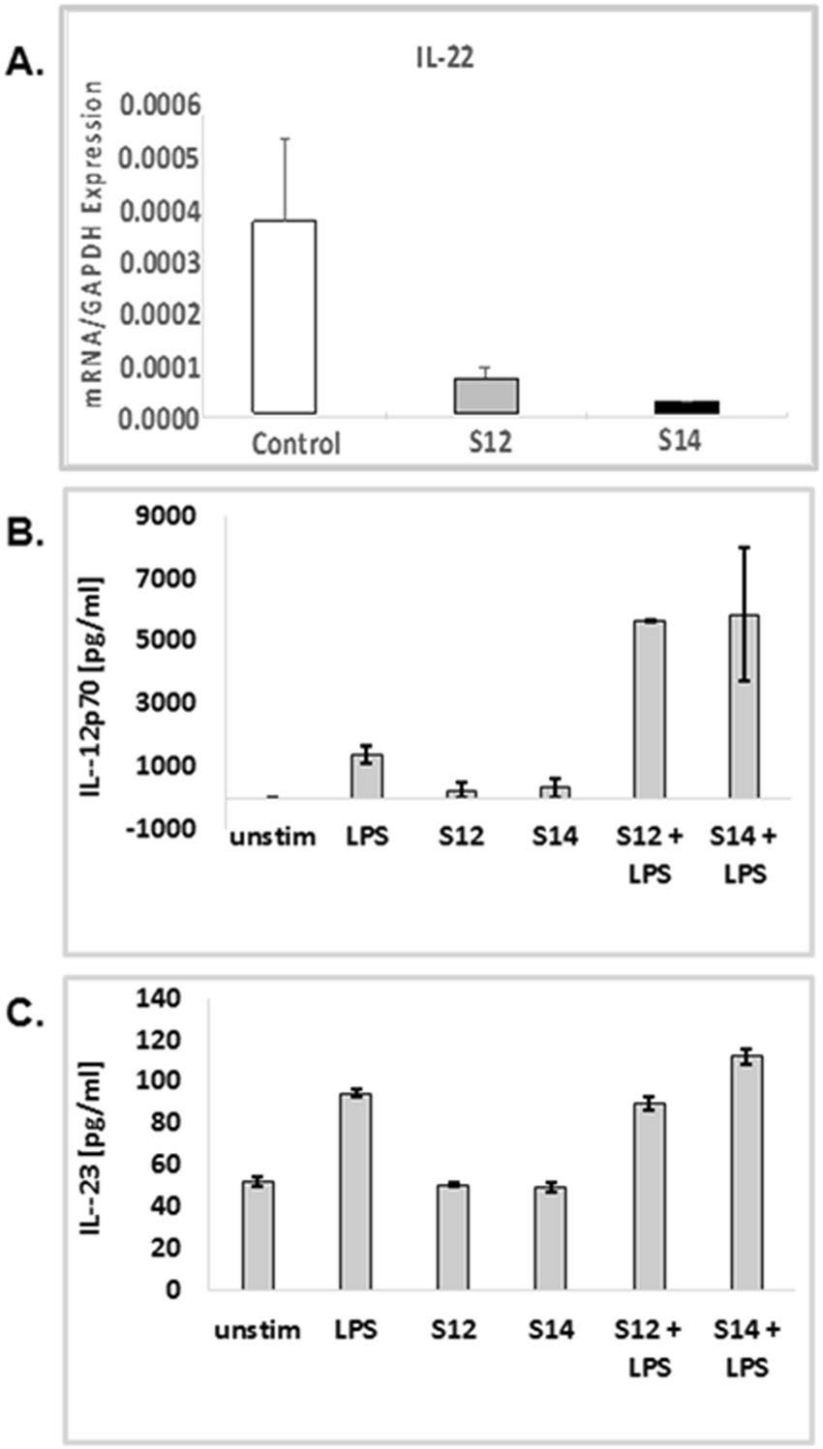
Solenopsin analogs S12 and S14 inhibit IL-22 expression. Compounds cause a significant decrease in IL-22 mRNA expression. The compounds also increase levels of IL-12 but no changes in IL-23 production by DC. Mouse DC were pre-incubated for 12 h with 10 µM compounds and then stimulated with LPS. IL-22 mRNA expression was measured by qRT-PCR. (**A**) Concentrations of IL-12 (**B**) and IL-23 (**C**) in the culture supernatants were determined by ELISA. Results are expressed as mean ± SD.

Since the solenopsin analogs were shown to decrease DC infiltration, and given DC are a source of IL-22, antiangiogenic IL-12 and proangiogenic IL-23, we wanted to investigate the effect of S12 and S14 on DC. Murine DC were pre-incubated with the compounds and then stimulated with a TLR4-ligand, LPS. The compounds significantly inhibited mRNA expression of IL-22 (Fig. 4A) and strongly induced IL-12p70 production in DC (Fig. 4B). This effect was not observed in IL-23 production (Fig. 4C).

### Solenopsin Analogs Downregulate TLR4 expression in Keratinocytes

**Figure 5:**
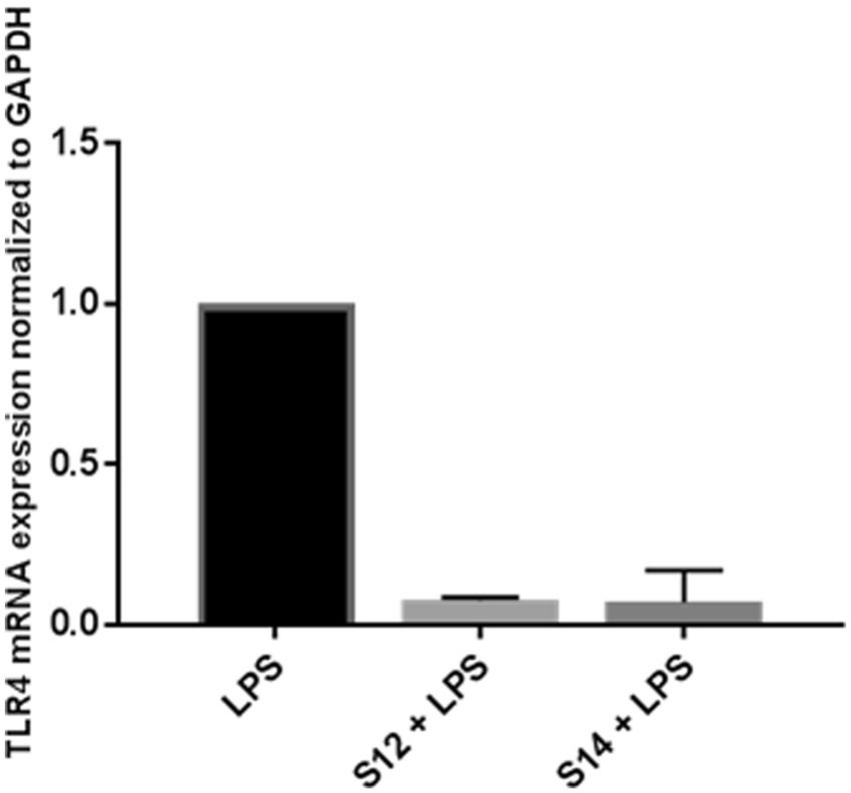
TLR4 in human HaCat keratinocytes treated with LPS, a canonical activator of TLR4. Treatment with the solenopsin derivatives S12 and S14 led to a significant decrease in TLR4 expression. Cells were treated with solenopsin analogs (in DMSO) and/or primed with LPS (10 µg/ml) for 24 h.

TLR4 serves as a co-receptor for S100A8 and S100A9 and has been shown to be a prominent mediator of both inflammation and tumorigenesis^10^. Given that S100A8 and S100A9 are downregulated by solenopsin analogs and that S100A8/A9 expression of TLR4 could mediate a positive feedback loop, we looked at the expression of TLR4 in human HaCaT keratinocytes treated with LPS, a canonical activator of TLR4. Treatment with the solenopsin derivatives S12 and S14 led to a significant decrease in TLR4 expression (Fig. 5).

## Discussion

Psoriasis is a common skin disorder that afflicts approximately 1% of the population^3, 21^. Increasing evidence of systemic involvement is suggested by epidemiologic association with other diseases, including coronary artery disease, obesity, and metabolic syndrome^21^. In recent years, targeting cytokines has become very popular for the treatment of severe psoriasis, with modalities targeting TNFα, IL-12, and, most recently, IL-17^22–25^. These modalities are reserved for the most severe cases, as they can result in systemic immunosuppression and are also costly.

Most cases of psoriasis are classified as mild or moderate and topicals are a mainstay of therapy. Topical corticosteroids remain the mainstay of therapy for the last 5 decades, with other topicals (retinoids, calcineurin inhibitors and vitamin D analogs) playing a relatively minor role in the management of psoriasis^26^. While effective in some cases, topical corticosteroids exhibit some negatives as well, including loss of efficacy, rebound, and skin atrophy. Therefore, alternative therapies are needed for the treatment of mild to moderate psoriasis.

Barrier disruption is a well-known clinical phenomenon in inflammatory and malignant skin disease. We have previously demonstrated that barrier disruption induces the potent angiogenic mediator VEGF, which contributes to chronic inflammation and angiogenesis^7^. Ceramides may also impact on the neuroendocrine environment of the skin in a manner to prevent excess inflammation and angiogenesis^27, 28^. The biochemical effectors of barrier disruption are not fully understood, but one candidate is a family of lipids known as ceramides, a class of fatty acid amides. Ceramides are synthesized from fatty acid precursors and degraded in part through amide hydrolysis. Ceramides have been demonstrated to have anti-inflammatory and antitumor properties. The beneficial effects of ceramides have been explained in part by the normalization of lipid rafts, which subsequently activates the tumor suppressor PTEN, as well as stimulating mitophagy^29^. Disordered ceramide synthesis has been shown to result in an inflammatory skin disorder in mice^8, 30, 31^.

In order to augment ceramide signaling, we synthesized a series of non-hydrolysable ceramide analogs that exhibit ceramide-like signaling, including normalization of lipid rafts and mitophagy^9, 32, 33^. Since these compounds, analogs of the ant venom compound solenopsin, lack amide groups, they cannot be hydrolyzed. We analyzed the effect of a topical 1% formulation of two analogs, S12 and S14, which we have previously found to be the most biologically-active derivatives.

In order to determine the mechanism of the anti-inflammatory activity of topical solenopsin derivatives, we performed gene array analysis on active- vs. vehicle-treated skins and looked for genes that were coordinately upregulated by both S12 and S14. The top most regulated genes were S100A8 and S100A9, which are downregulated by the derivatives. These two genes form a complex called calprotectin that protects against reactive oxygen stress, chelates zinc, and has antimicrobial properties. Of interest, calprotectin is upregulated in virtually every murine model of psoriasis, including this model, imiquimod-induced inflammation, and loss of selective AP-1 subunits from the epidermis (c-Jun/JunB), and is highly expressed in human psoriasis, but not atopic dermatitis^1, 11, 34^. Our derivatives also downregulate TLR4, a co-receptor for calprotectin. Examination of the promoter sequences of S100A8, S100A9, and TLR4 reveal that these genes are regulated by NFκB and AP-1^35, 36^. It should also be noted that both S100A8 and S100A9 are highly expressed in vascular atheroma of atherosclerosis that is highly associated with psoriasis^37, 38^.

Traditionally, psoriasis has been thought to be mediated by CD4 lymphocytes. However, recent findings have pointed towards a distinct role of CD8 lymphocytes in psoriasis. First, CD8 lymphocytes have been found to be more abundant in psoriatic epidermis than CD4 lymphocytes^39^. Second, subsets of CD8 lymphocytes have been shown to be of the Tc22 lineage, which is a major producer of IL-22 in the skin.

Both S12 and S14 also upregulated the AP-1 transcriptional regulators FosB and ATF3 in gene arrays. These genes have previously been demonstrated to be downregulated in non-lesional psoriatic skin compared with normal skin, thus suggesting that even non-lesional skin in psoriasis patients may have disturbances in ceramide metabolism and that this disturbance may predispose skin to psoriatic inflammation^8, 40^.

Finally, solenopsin derivatives modulate DC to produce IL-12 rather than IL-23. IL-12 has been shown to be anti-angiogenic and, more recently, has been shown to have anti-psoriatic activity on its own^41^. Thus, it is likely that solenopsin analogs, by restoring ceramide signaling, restore normal skin homeostasis potentially through an anti-inflammatory AP-1/IL-12 pathway.

Our findings have direct relevance to human psoriasis. S100A8 is upregulated by some of the current treatments for human psoriasis, namely glucocorticoids and UVA^40, 42, 43^. We believe this to be a compensatory upregulation of S100A8 and related molecules in response to treatment, which may be a mechanism of resistance to anti-psoriatic therapy. S100A8 may thus serve as a biomarker for the effectiveness of anti-psoriatic drugs, and combination therapies with solenopsin analogs and other modalities for psoriasis, such as glucocorticoids and UV light, may lead to long-term remission. Further studies of these analogs as anti-psoriatic drugs are warranted in humans.

## Materials and Methods

### Mouse Model

K5tTA and TetosTek/Tie2 mice (on a CD1 outbred background) were crossed to generate double transgenic KC-Tie2 mice as described previously^44^. These mice spontaneously develop an inflammatory skin phenotype that phenocopies human psoriasis, including an ~6-fold increase in epidermal thickness (acanthosis), infiltrating CD4+ and CD8+ T cells, and a dense cutaneous myeloid cell presence (CD11c+, CD11b+ and F4/80+), along with the development of munro-like microabscesses containing GR1+ neutrophils^44^. The skin resembles human psoriasis at the transcriptomic^45^ and proteomic levels^46^. The cutaneous phenotype is reversible following broad immunosuppression using cyclosporine A (CsA)^44^ and inhibition of clinically efficacious targets, including TNFα, IL-23 or antigen cell depletion^20–22^. Importantly, KC-Tie2 mice fail to respond to drugs to which psoriasis patients have failed to respond^47, 48^. Thus, the KC-Tie2 model provides a critical tool for testing efficacy of responsiveness to potential anti-psoriasis drugs.

Adult male and female KC-Tie2 mice with established skin disease were treated with the compounded S12 (n = 9) or S14 (n = 9) analogs or control vehicle cream (n = 8) applied to their dorsal skin 1x/day for 28 days. At the completion of the experiment, mice were euthanized and dorsal skin harvested and processed as described previously^44^ for histological, immunostaining and transcriptomic analyses.

Animal protocols were approved by the Case Western Reserve University Institutional Animal Care and Use Committee (IACUC; Cleveland, OH) and were consistent with guidelines issued by the American Association for Accreditation of Laboratory Animal Care.

### Histological and immunostaining analyses

Dorsal skin was collected and processed as described previously for histology and immunohistochemistry^44^. Formalin-fixed, paraffin-embedded skin was sectioned and stained with haematoxylin and eosin (H&E) using standard techniques as described^44^. Fresh frozen skin was sectioned and stained, as previously published^44, 49^, using antibodies targeting the following proteins: CD4 (clone RM4-5, Cat.#550280), CD8a (clone 53-6.7, Cat.#550281), CD11c (clone HL3, Cat.#550283; all BD Pharmingen; San Jose, CA) and F4/80 (clone BM8, Cat.#14-4801, eBioscience; San Diego, CA).

Epidermal thickness (acanthosis) measurements and immune cell quantification in dorsal skin sections was completed using microscopic images collected from the stained sections using interactive image analyses approaches as described previously^44^.

### Gene array analysis

Dorsal skin samples from KC-Tie2 mice treated with control vehicle, S12 or S14 were excised, snap-frozen and submitted to the Emory Integrated Genomics Core for RNA extraction, quality analysis and gene expression assay. Gene expression analysis was performed using an Affymetrix GeneChip^TM^ Microarray: Mouse Gene ST 2.0 and Gene Expression Module of GenomeStudio Software (v2011.1, Illumina).

### Generation of murine bone marrow-derived myeloid dendritic cells (BMDCs)

Murine dendritic cells (DC) were generated from bone marrow cells according to the method of Guenova *et al*.^50^. Tibiae and femurs from naive mice were removed under sterile conditions. Both ends of the bones were cut off and bone marrow was collected from the medullary cavities by flushing with a PBS-filled syringe. Bone marrow was disrupted by pushing the clumps through 40 μm cell strainers using the rubber tip of a syringe plunger. Cell suspensions were centrifuged and the cell pellets were resuspended in 500 μl hypotonic ACK Lysing Buffer and incubated for 3 min to lyse the erythrocytes. Cells were centrifuged, washed with medium, and counted.

Bone marrow cells were resuspended at 2 × 10^5^ cells/ml in DC culture medium containing 200 U/ml rmGM-CSF (Cat.#415-ML, R&D) and 10 ml were plated into 100 × 15 mm plastic petri dishes (Cat# 351029, BD). On day 3, an additional 10 ml DC culture medium containing 200 U/ml rmGM-CSF (R&D) were added and, at day 6, half of the culture supernatant was removed and centrifuged. The cell pellet was resuspended in 10 ml DC culture medium containing 200 U/ml rmGM-CSF and transferred back into the plate. At day 7, BMDCs (non-adherent cells) were harvested and used for stimulation. Cells were treated with vehicle, S12 or S14 compounds (5 µM or 10 µM) overnight and, at day 8, DC were further stimulated with lipopolysaccharide (LPS; 100 ng/ml, from *Salmonella minnesota* R595, Alexis Biochemicals, Lausanne, Switzerland) for 24 h.

Supernatants were collected for ELISA. For FACS analysis, BMDCs were harvested and labeled with appropriate antibodies to measure the expression of activation markers and costimulatory molecules. Cytokines were detected by ELISA using commercially available kits for determination of IL-12p70 (BD Biosciences, Cat.#555256) and IL-23 (eBioscience, Cat.#88-7230).

### TLR4 Assessment

Human keratinocyte (HaCaT) cell line was obtained from UAB Skin Disease Research Center and propagated in monolayer in Dulbecco’s Modified Eagle’s Medium supplemented with 10% heat-inactivated fetal bovine serum, 100 μg/ml penicillin and 100 μg/ml streptomycin from Life Technologies (Carlsbad, CA). The cells were maintained in 5% CO_2_ in a humidified incubator at 37 °C. Cells were treated with different concentrations of solenopsin analogs (in DMSO) and/or primed with LPS (10 µg/ml) for 24 h.

Total RNA was extracted from HaCaT cells using TRIzol (Life Technologies, Carlsbad, CA). The concentration of total RNA was determined by measuring the absorbance at 260 nm using a Bio-Rad Smart Spec spectrophotometer (Hercules, CA). cDNA was synthesized from 1 µg RNA using Reverse Transcriptase kit (Bio-Rad, Hercules, CA) according to kit instructions. Using iQ^TM^ SYBR Green Master Mix (Bio-Rad, Hercules, CA), cDNA was amplified by qPCR using human TLR4 primers with a Bio-Rad MyiQ thermocycler and SYBR Green detection system (Bio-Rad, Hercules, CA). The standard PCR conditions were 95 °C for 10 min followed by 40 cycles at 95 °C for 30 s, 60 °C for 30 s, and 72 °C for 30 s. The expression level of TLR4 was normalized to the expression level of GAPDH in each sample. Relative mRNA expression for duplicate samples was calculated using the ΔΔCT method.

Human TLR4 Forward: AAGCCGAAAGGTGATTGTTG

Human TLR4 Reverse: CTGAGCAGGGTCTTCTCCAC

### IL-22 mRNA assessed by qRT-PCR

IL-22 mRNA was assessed by qRT-PCR as previously described by Fritz *et al*. with primers purchased from Applied Biosystems - primers chosen to span intro-exon^51^.

### Statistics

Comparisons between groups for acanthosis and immune cell density were performed using an unpaired, two-tailed, unequal variance Student’s t-test. Probability values less than 0.05 were considered significant. Data are presented as mean ± SEM.

### Statistics for GeneChip^TM^ Analysis

Probe level intensity values were extracted and quantile normalization was performed using the Gene Expression Module of the GenomeStudio Software (v2011.1, Illumina). Principle Component Analysis (PCA) and unsupervised hierarchical clustering were used to assess the variation in the expression profiles. The Significance Analysis of Microarrays (SAM; http://statweb.stanford.edu/~tibs/SAM/) was used to derive a refined list of genes most affected by S12 and S14 compared to the vehicle control.

### Data Availability

All data generated or analyzed during this study are included in this published article (and its Supplementary Information files).

## Electronic supplementary material

Figures S1 and S2
