# Supplementary material for: Evidence for biochemical barrier restoration: Topical solenopsin analogs improve inflammation and acanthosis in the KC-Tie2 mouse model of psoriasis
Source: Sci Rep. 2017 Sep 11;7:11198. doi: 10.1038/s41598-017-10580-y (PMC5593857; doi:10.1038/s41598-017-10580-y)
Supplement: Supplementary file 1 — Figures S1 and S2 [file 41598_2017_10580_MOESM1_ESM.pdf]

Evidence for biochemical barrier restoration: Topical solenopsin analogs improve inflammation and acanthosis in the KC-Tie2 mouse model of psoriasis

Jack L. Arbiser[1,2] *, Ron Nowak[3], Kellie Michaels[3], Yuliya Skabytska[4], Tilo Biedermann[4], Monica J. Lewis[5], Michael Y. Bonner[1], Shikha Rao[1], Linda C. Gilbert[1,2], Nabiha Yusuf[5], Isabella Karlsson[1], Yi Fritz[3] and Nicole L. Ward[3]

Department of Dermatology, Emory University School of Medicine, Atlanta, GA, 30322[1], Veterans Affairs Medical Center, Decatur, GA 30322[2], Case Western Reserve University, Cleveland, OH 44106[3], Department of Dermatology and Allergology
Technische Universität München, Germany[4], and Department of Dermatology, University of Alabama, Birmingham, AL 35294[5]

*Corresponding author:
Jack L. Arbiser

Department of Dermatology, Emory University School of Medicine

WMB 5309, 101 Woodruff Circle

Atlanta, GA 30322

Tel (404) 727-5063

Fax (404) 727-0923

Email: jarbise@emory.edu

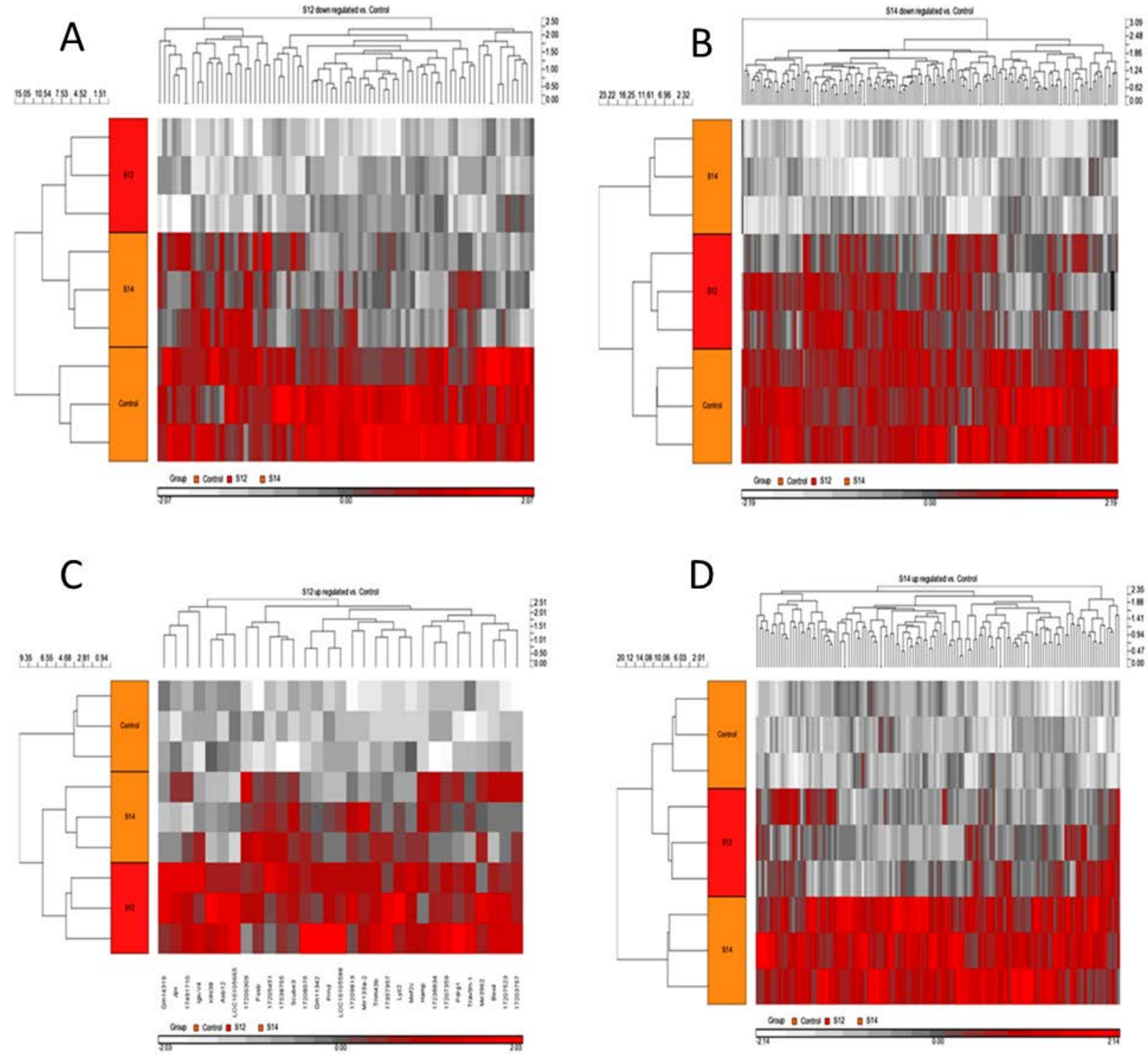
A
S12 down regulated vs. Control
B
S14 down regulated vs. Control
C
S12 up regulated vs. Control
D
S14 up regulated vs. Control
Group
Control
S12
S14

Figure S1

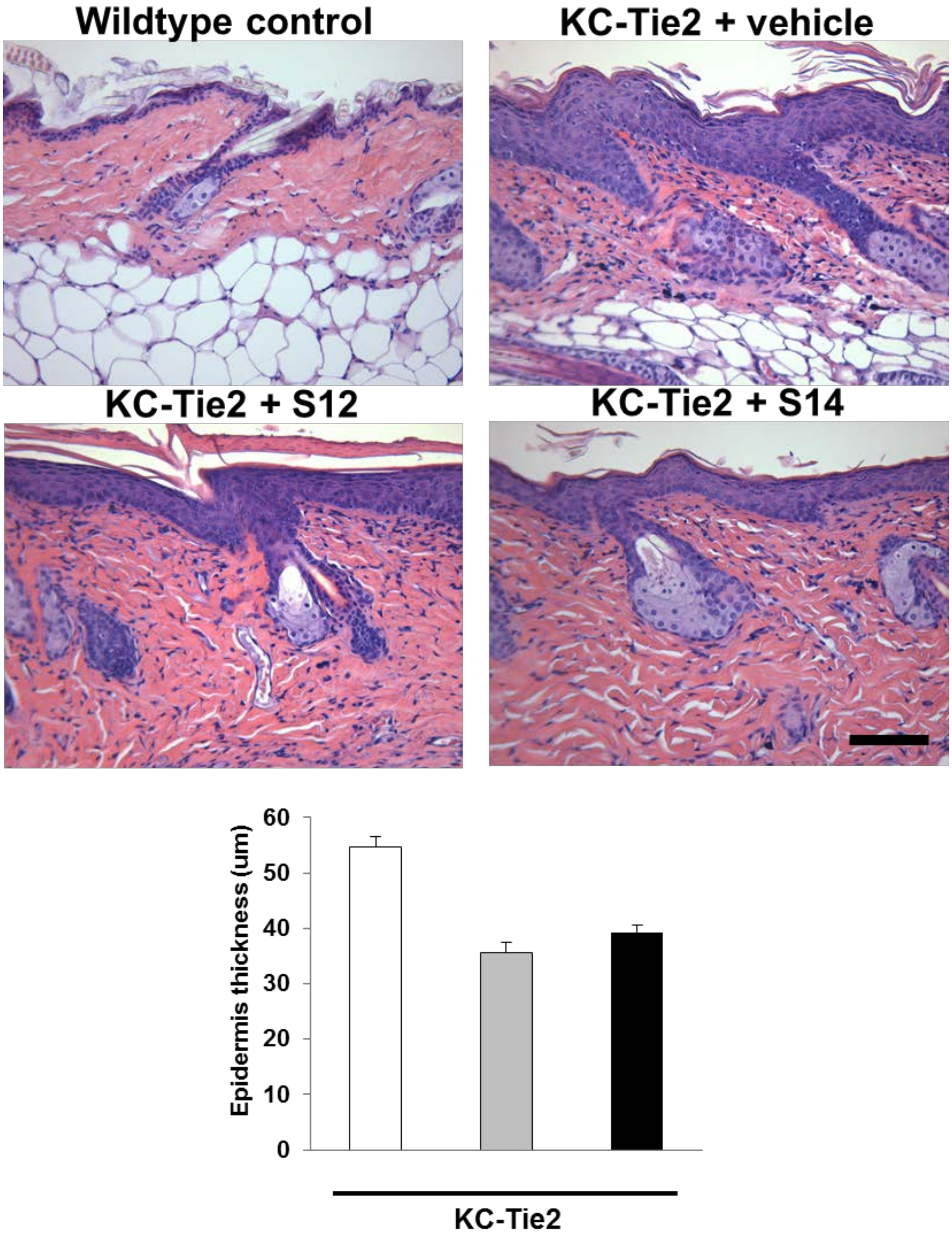

Wildtype control
KC-Tie2 + vehicle
KC-Tie2 + S12
KC-Tie2 + S14
Epidermis thickness (um)
0
10
20
30
40
50
60
KC-Tie2

Figure S2

Figure S1. **Skin from vehicle, S12, and S14 treated KC-Tie2 mouse skin was subjected to gene array analysis and heat map analysis to identify upregulated and downregulated transcripts.** Gene array results are arranged to show the relative gene expression levels between experimental groups. On each heat map, the experimental group with the largest gene expression change (shades of red) is at the bottom and the group with the least amount of change (shades of white to gray) across the microarray is on the top. Genes are arranged horizontally based on clusters of similar expression levels. A. S12 downregulated vs. Control. B. S14 downregulated vs. Control. C. S12 upregulated vs. Control. D. S14 upregulated vs. Control.

Figure S2. Treatment of KC-Tie2 mice with S12 and S14 results in decreased epidermal thickness (acanthosis). Archival nontransgenic skin as Wildtype control is included as a comparison. Representative images of H&E-stained dorsal skin sections from KC-Tie2 mice following treatment with S12, S14 or vehicle cream. (B). Quantification of epidermal thickness (µm) of H&E-stained dorsal skin sections of vehicle treated (n=8), S12 (n=9) and S14 (n=9) KC-Tie2 mice. Values shown represent the mean ± SEM. Data were analyzed using a Student's t-test. P values are as indicated. Scale bar = 50µm.
